# A Silent Killer in the Waters: A Report of Two Cases of Vibrio vulnificus Bacteremia

**DOI:** 10.7759/cureus.103345

**Published:** 2026-02-10

**Authors:** Carlos Fagundo, Junaid Saleh-Esa, Adriana Hernandez, Parul Aneja

**Affiliations:** 1 Internal Medicine, BayCare Health System, Tampa, USA; 2 Infectious Diseases, BayCare Health System, Tampa, USA

**Keywords:** climate change and its effect on life and health, flood water exposure, necrotizing fasciitis, vibrio bacteremia, vibrio vulnificus

## Abstract

In this case series, we report two unusual cases of *Vibrio* bacteremia due to exposure to floodwater after Hurricane Helene. This presentation is particularly alarming in light of growing evidence that warming ocean temperatures are contributing to stronger hurricanes, which, in turn, lead to more extensive flooding and storm surges, a trend that creates ideal conditions for the spread of waterborne pathogens such as *Vibrio vulnificus*. Previously, within the humid subtropical region, only a handful of cases have been documented over the past 20 years. With the increasing number of cases, it is now crucial to highlight the swift diagnosis and treatment of infection before the development of sequelae or mortality. In our two-case series, both patients initially presented with nonspecific symptoms of *Vibrio* bacteremia, with common waterborne exposure. Both cases were ultimately successfully treated with conventional therapy with doxycycline and a third-generation cephalosporin, and with only one patient requiring surgical debridement. Consequently, it is important to acknowledge the clinical signs and symptoms of *Vibrio* bacteremia, as a lack of prompt diagnosis and treatment can lead to severe complications or even death.

## Introduction

We present two patients who developed rapidly progressive lower extremity cellulitis and *Vibrio vulnificus* bacteremia due to Hurricane Helene floodwater exposure. Their parallel courses, one salvaged with medical therapy, the other requiring timely surgical debridement, illustrate the importance of prompt identification and treatment. The cases are unique in documenting the value of early microbiology notification; demonstrating limb preservation through prompt therapy with doxycycline plus ceftazidime, consistent with current Centers for Disease Control and Prevention guidance; and adding real-world evidence that severe *V. vulnificus *disease can occur after exposure to floodwaters, reinforcing the need for heightened clinician awareness as extreme weather events become more frequent [1-4].

## Case presentation

Case 1

**Figure 1:**
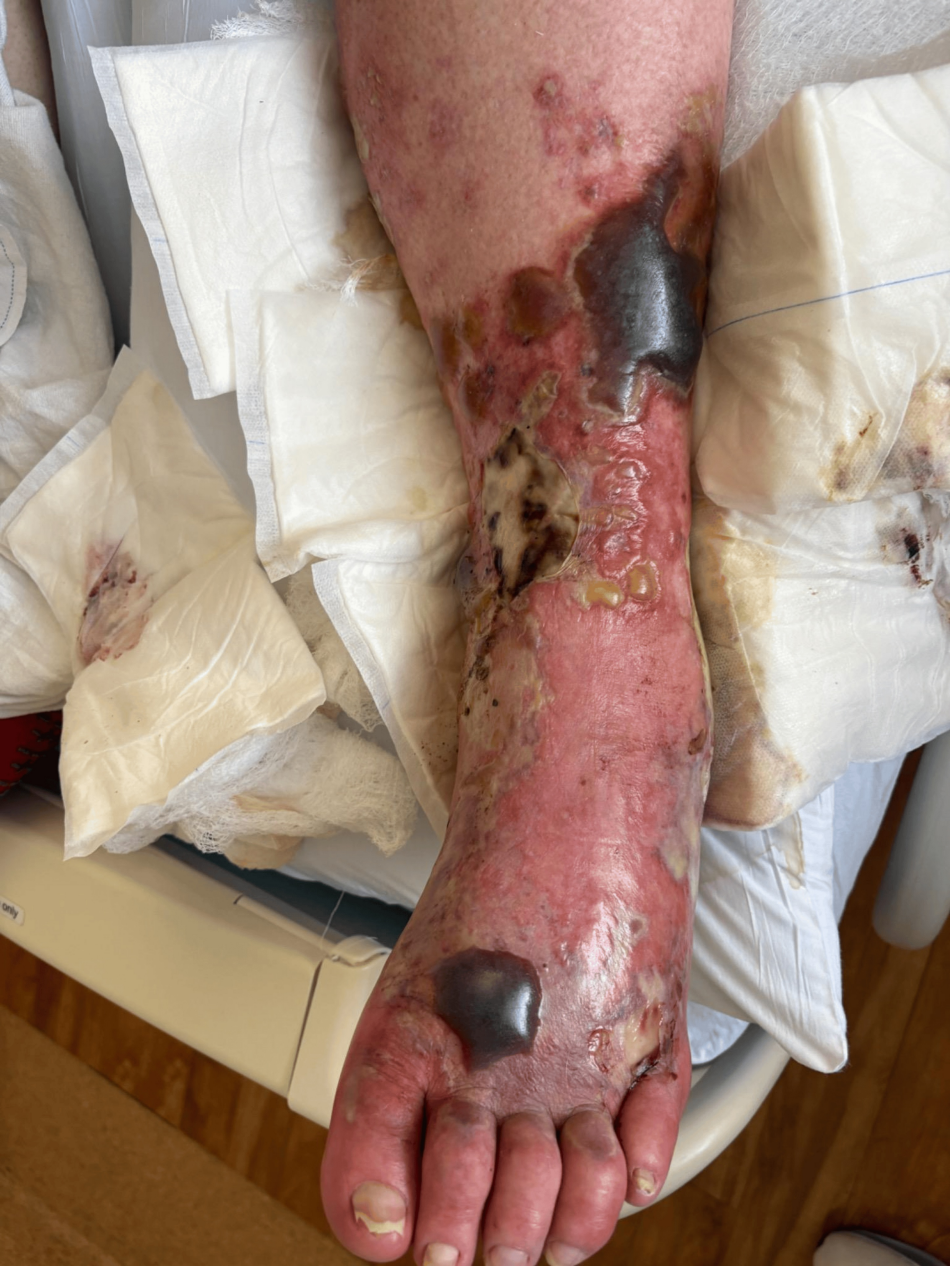
Patient 1: day two of hospital admission and day four of infection, very similar to the initial presentation reported by the patient.

**Figure 2:**
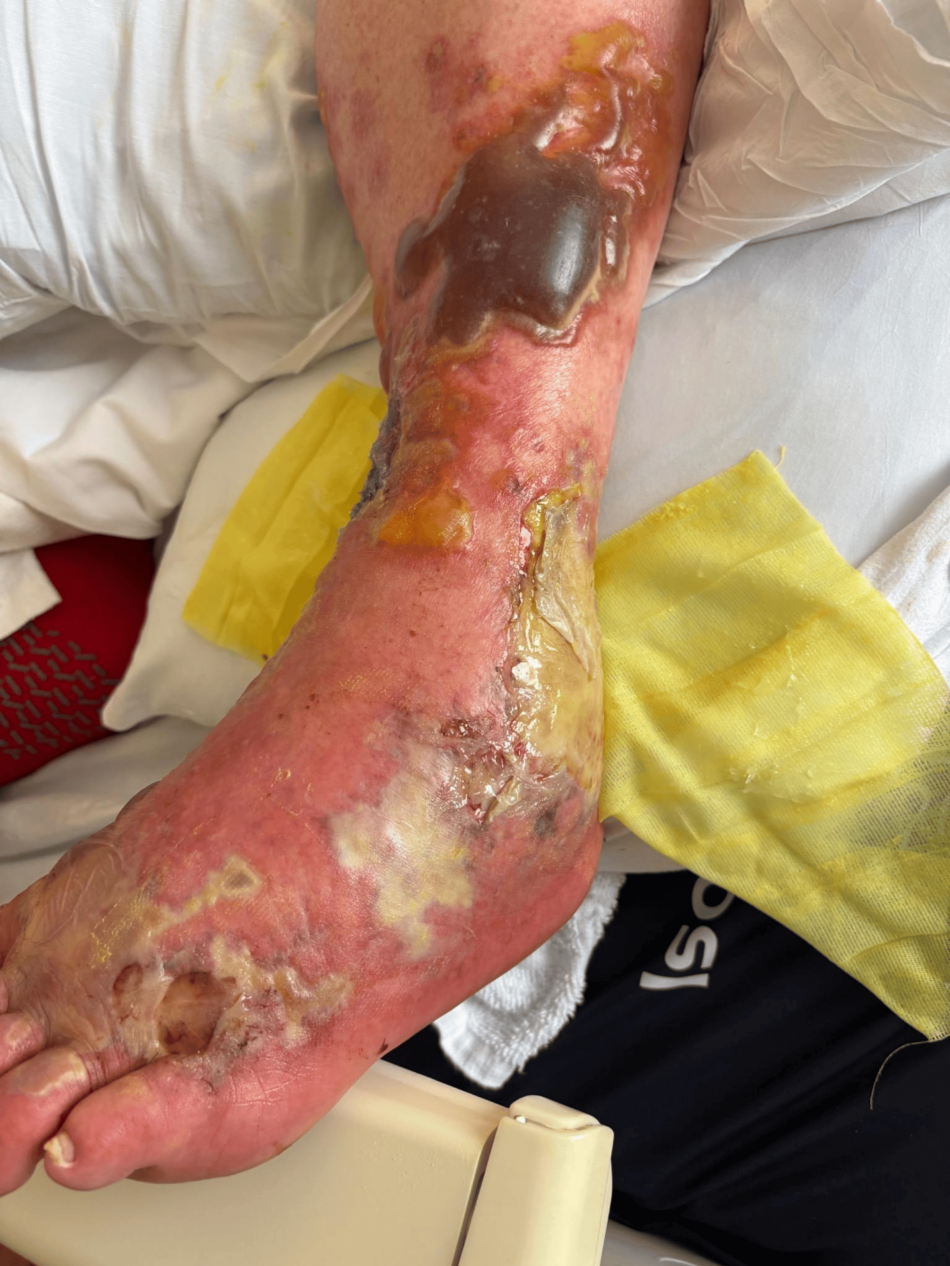
Patient 1: day three of hospital admission, with marked progression of liquefactive necrosis.

**Figure 3:**
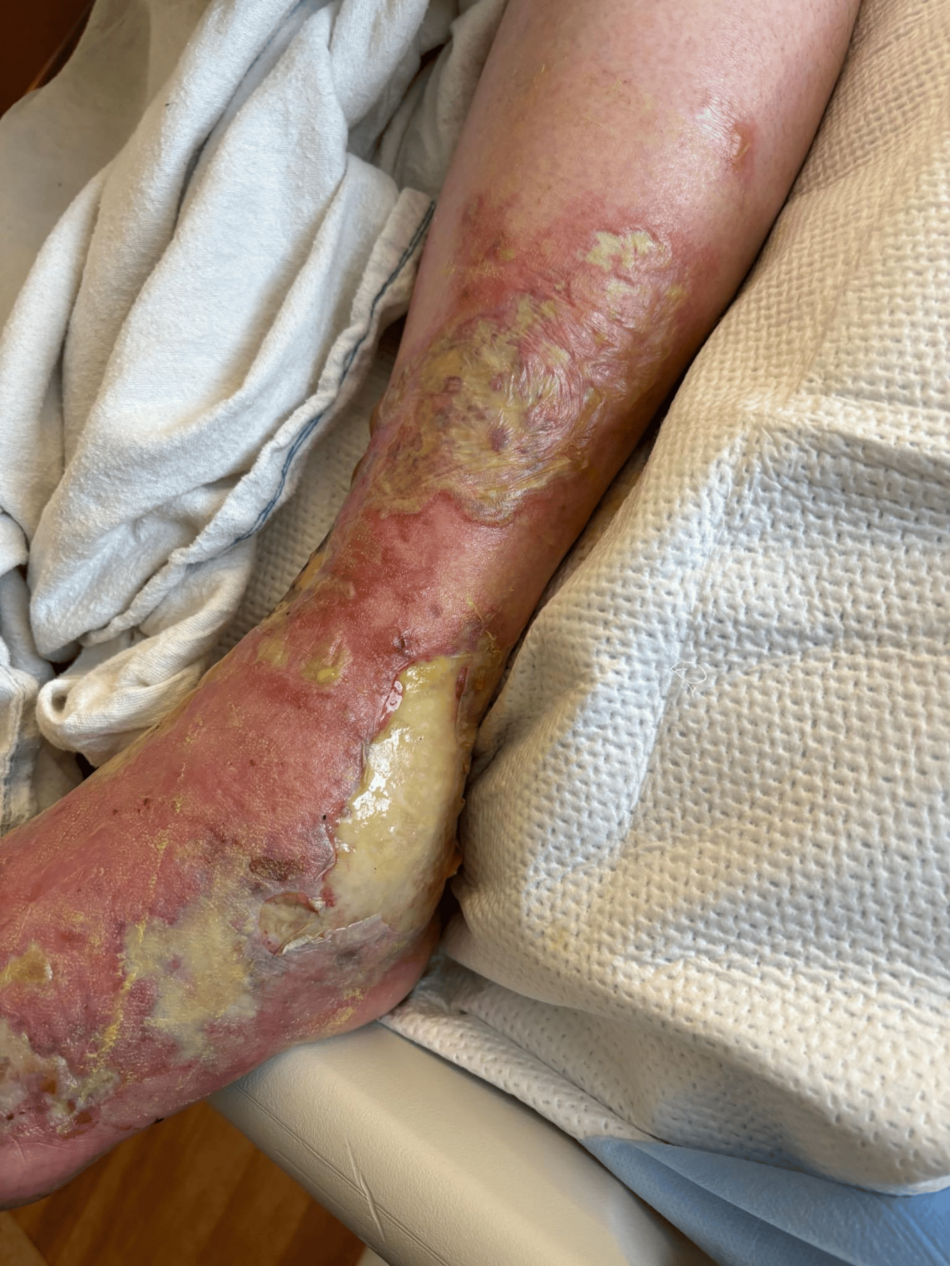
Patient 1: day four of admission, with resolution of bullae.

**Figure 4:**
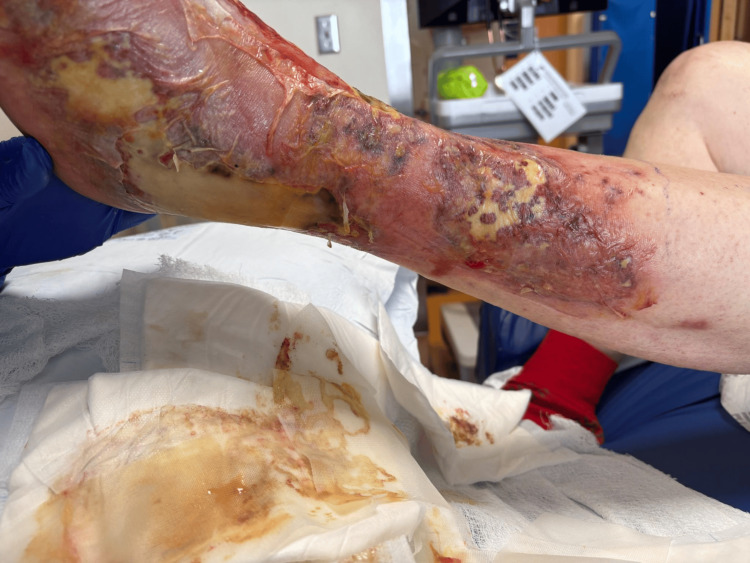
Patient 1: day nine, with no further decline in presentation.

A 67-year-old male presented to the emergency room after a ground-level fall during a hurricane-related flood. He had no reported past medical history. His legs were exposed to brackish flood waters and sustained multiple insect bites. He attempted to take care of the lower extremity wounds using supportive measures at home by applying topical hydrocortisone cream. He did not experience relief from his symptoms and sought care at our facilities. Initial physical examination revealed a body temperature of 98.2 °F, respiration rate of 22 breaths per minute, blood pressure of 154/74 mmHg, and heart rate of 58 beats per minute. Figure 1 shows the wound on day two of hospital admission. The left lower extremity had multiple hemorrhagic bullae with several areas of rupture. Figure 2 shows the progression of suppuration. On day four of hospital admission, all hemorrhagic bullae ruptured (Figure 3). His wounds started to show major resolution on hospital day nine (Figure 4).

The patient’s laboratory findings showed an elevated white blood cell count, absolute neutrophil count, and 42% bands (Table 1). Further laboratory studies showed elevated creatinine, blood urea nitrogen, and elevated liver enzymes (Table 1).

**Table 1:** Laboratory findings of Patient 1 at admission.

Parameter	Patient’s value	Reference range
White blood cell count	17.1 × 10⁹/L	4.0–11.0 × 10⁹/L
Absolute neutrophil count	15.7 × 10⁹/L	1.5–7.5 × 10⁹/L
Bands	42%	0–3%
Creatinine	2.89 mg/dL	0.6–1.2 mg/dL
Blood urea nitrogen	45 mg/dL	7–20 mg/dL
Alanine aminotransferase	82 U/L	7–56 U/L
Aspartate aminotransferase	157 U/L	10–40 U/L

**Figure 5:**
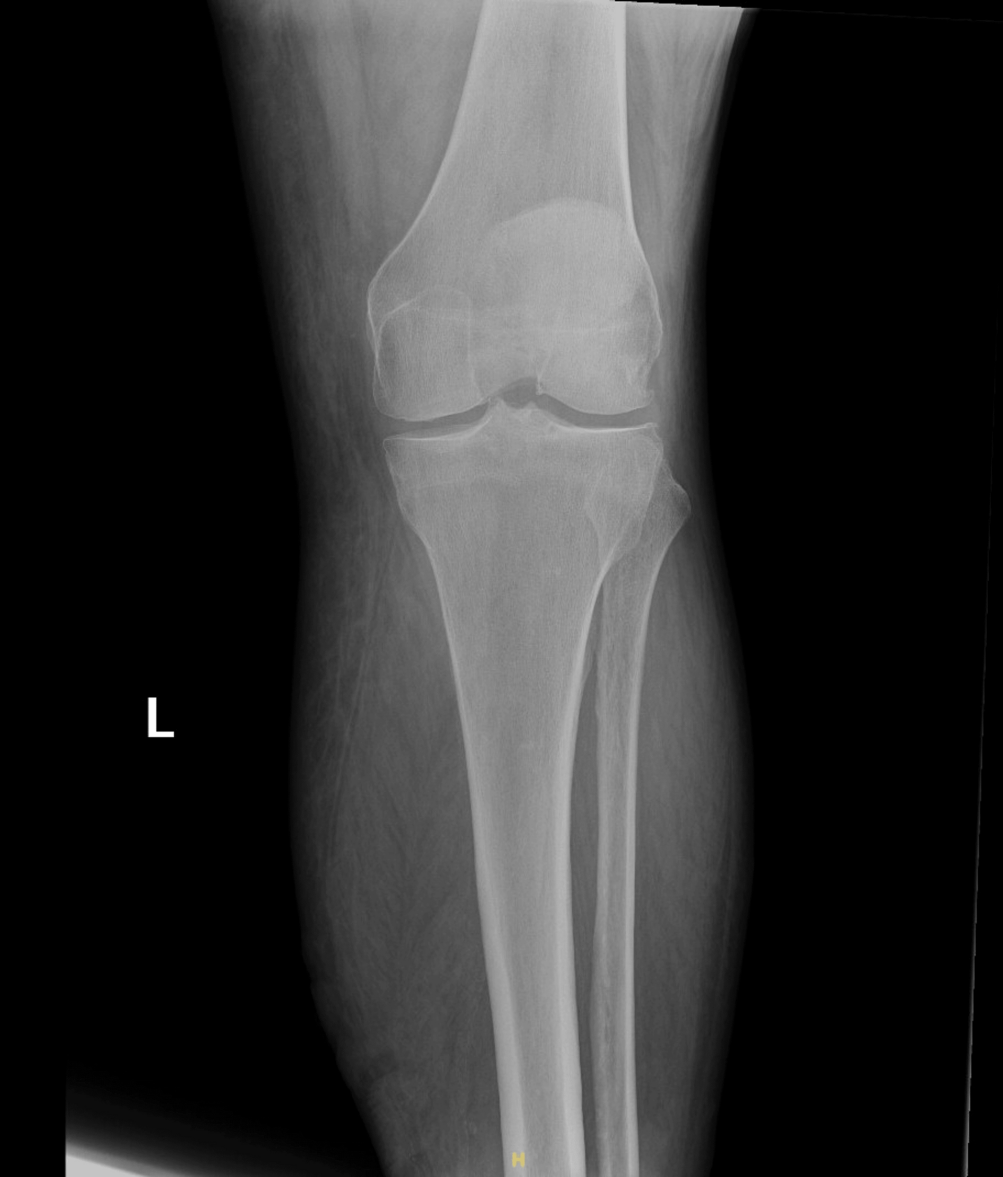
X-ray of the left tibula and fibula shwoing edematous and irregular soft tissues at the left leg and ankle.

**Figure 6:**
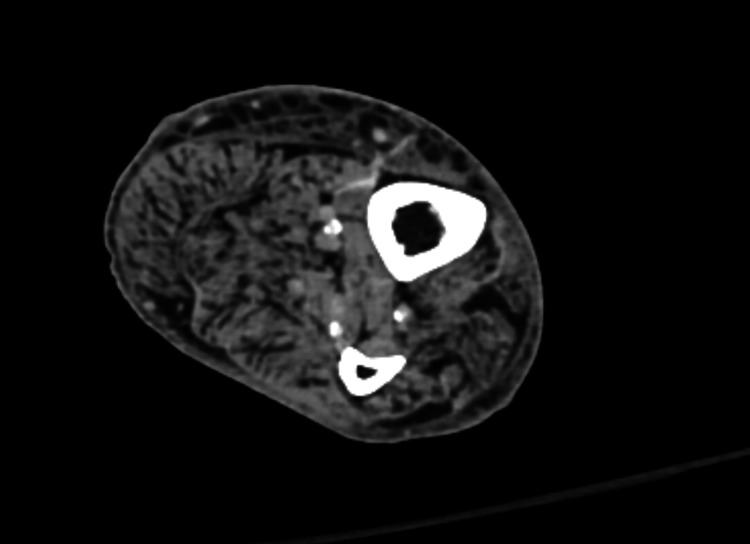
CT of the left lower extremity with contrast showing grossly edematous subcutaneous soft tissues at the distal half of the left leg. No subcutaneous emphysema. No organized fluid collection identified to suggest abscess.

A CT scan of the chest, abdomen, and pelvis identified peripherally calcified fluid in the right scrotal sac. Collections were likely unrelated to his fall. The left leg, ankle, and X-ray imaging revealed diffuse soft tissue edema without fractures or subcutaneous emphysema (Figure 5). A CT scan of the lower left extremity corroborated significant subcutaneous soft tissue edema (Figure 6). No subcutaneous emphysema or organized fluid collections were identified.

Given his recent exposure to brackish water and the elevated white blood cell count, the decision was made to admit the patient for inpatient care to closely monitor his condition. Initially, the patient was thought to have cellulitis, but there was no concern for necrotizing fasciitis. The patient was started on vancomycin, ceftazidime, and metronidazole. Blood cultures were collected before administering antibiotics. Cultures collected at admission showed microbial growth, and the preliminary blood culture reported Gram-negative rods. At that time, doxycycline was added while awaiting final identification and antimicrobial susceptibility results. Culture identification matrix-assisted laser desorption/ionization time-of-flight protocol was positive for growth of *V. vulnificus*. Sensitivity was established through Vitek 2 (bioMérieux, Marcy l'Étoile, France). On hospital day two, general surgery was consulted for debridement. Due to extensive tissue involvement, they consulted orthopedic surgery to assess if the patient would benefit from amputation. Amputation was discussed as a possible option. The patient declined amputation at that time, opting to continue with medical management. The patient experienced a prolonged hospital course due to the extent of soft tissue involvement and the need for close monitoring. Despite initial concerns for limb loss, his condition gradually improved with aggressive antimicrobial therapy and supportive care. He was ultimately discharged to a rehabilitation facility in stable condition, where he continued to recover while retaining full use of the affected limb.

Case 2

A 53-year-old male presented to the emergency room. He was sent from an urgent care facility due to not feeling well and a swollen right lower extremity. He had a past medical history of lower extremity deep vein thrombosis and morbid obesity. He had a prior chronic wound on his right lower extremity (highlighting an immunocompromised status) and noticed worsening redness and a new onset of yellow drainage from the wound, which prompted him to seek care at an urgent care facility. At that time, he was noted to be tachycardic and was sent to our emergency room for further care. He reported that he had been cleaning debris at his home after flooding, and pulling carpet exposed to floodwater from Hurricane Helene.

Initial physical examination revealed a body temperature of 102.9 °F, respiration rate of 22 breaths per minute, blood pressure of 132/63 mmHg, and heart rate of 116 beats per minute. The right lower extremity had two open puncture-like wounds on the anterior surface with erythema, purulent drainage, and bruising. His leg was warm to the touch.

The patient’s laboratory findings showed an elevated white blood cell count, absolute neutrophil count, and elevated lactic acid level (Table 2).

**Table 2:** Laboratory findings of Patient 2 at admission.

Parameter	Patient’s value	Reference range
White blood cell count	13.9 × 10⁹/L	4.0–11.0 × 10⁹/L
Absolute neutrophil count	11.2 × 10⁹/L	1.5–7.5 × 10⁹/L
Lactic acid	2.3 mmol/L	0.5–2.2 mmol/L

**Figure 7:**
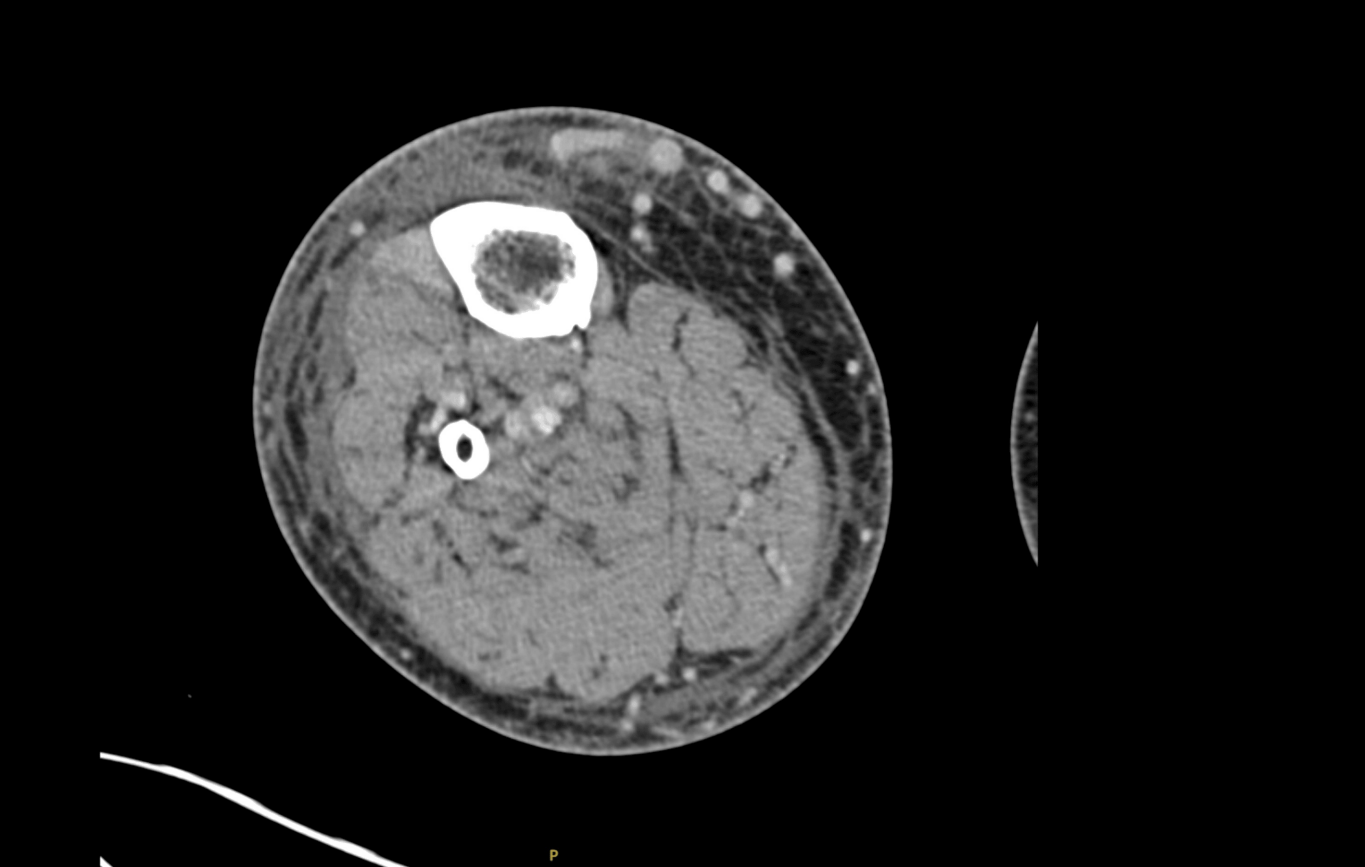
Lower extremity CT with contrast of the right lower extremity showing lower soft tissue edema and cellulitis without drainable fluid collection and no findings of osteomyelitis.

Imaging studies performed on arrival revealed no acute traumatic injuries. Upon the patient’s arrival, blood cultures were taken before antibiotic administration. A CT scan of the right lower extremity revealed skin thickening and edema within the subcutaneous tissues, increasing distally (Figure 7). Extensive varicosities were noted within the soft tissues, and there was superficial fascial fluid. No drainable fluid collections were identified, and there were no signs of osteomyelitis.

**Figure 8:**
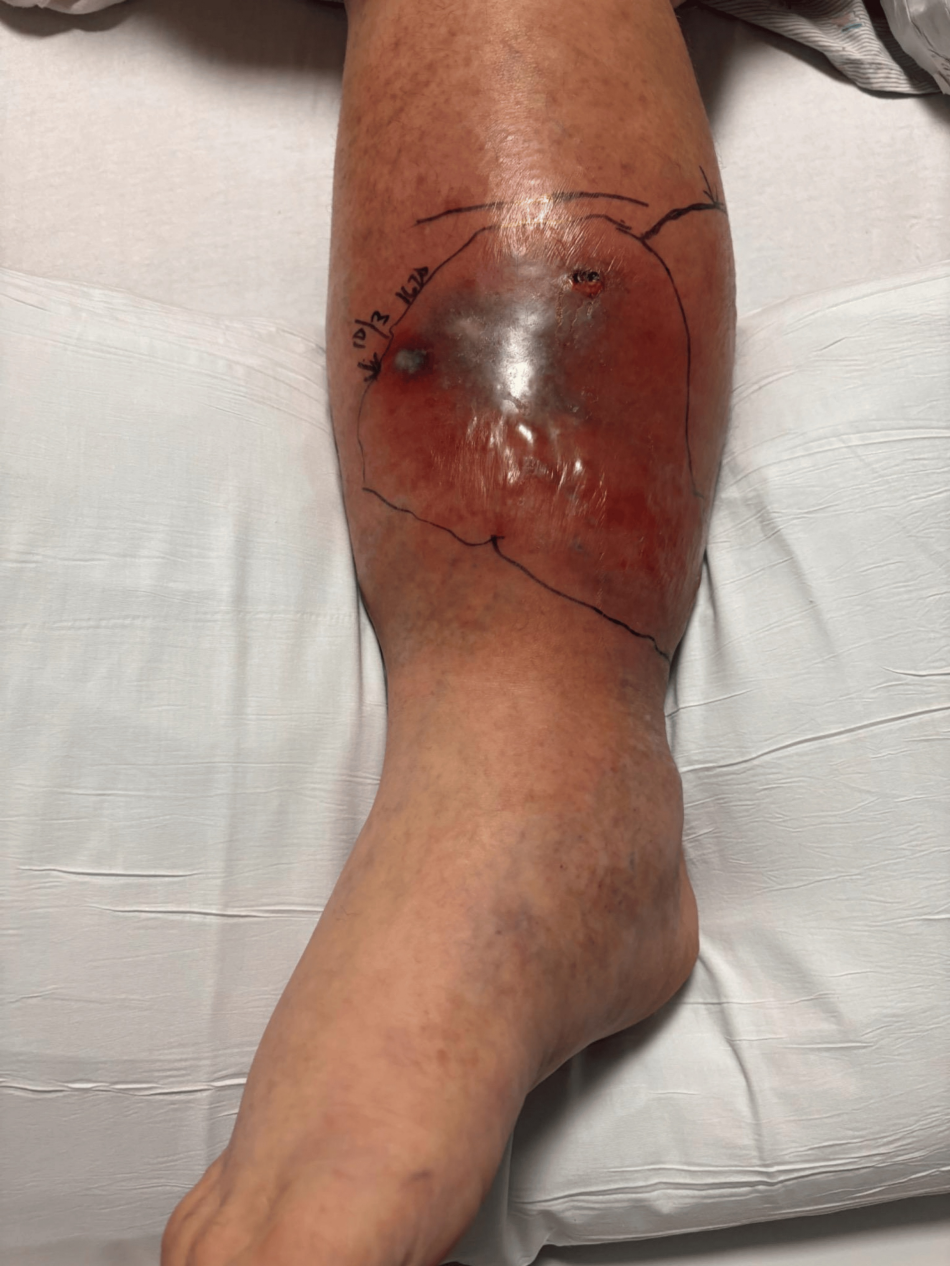
Patient 2: day one, initial presentation of the extremity at the time of hospital admission. Note minor bullae formation.

**Figure 9:**
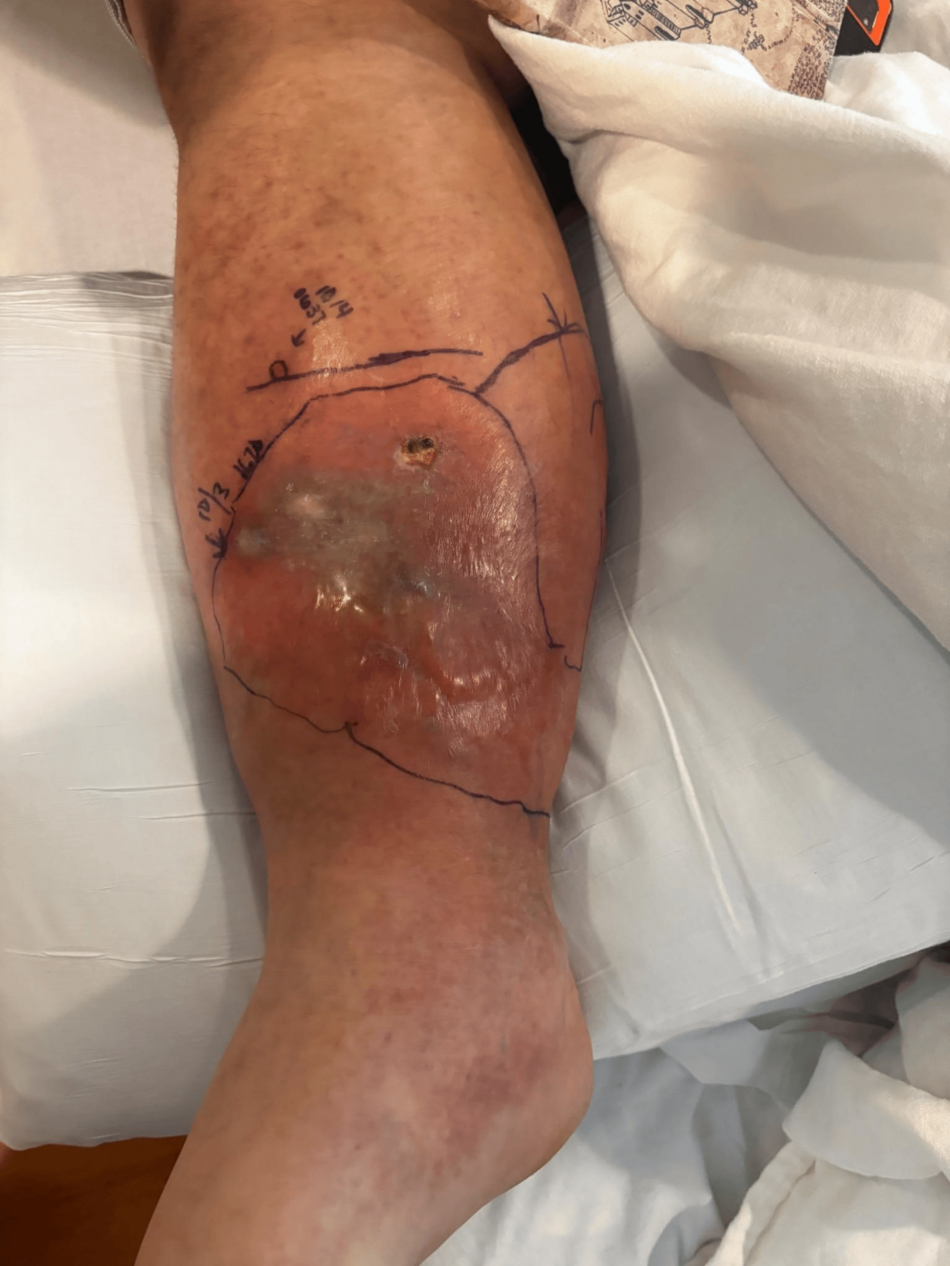
Patient 2: day two of hospitalization, with improvement in localized edema.

**Figure 10:**
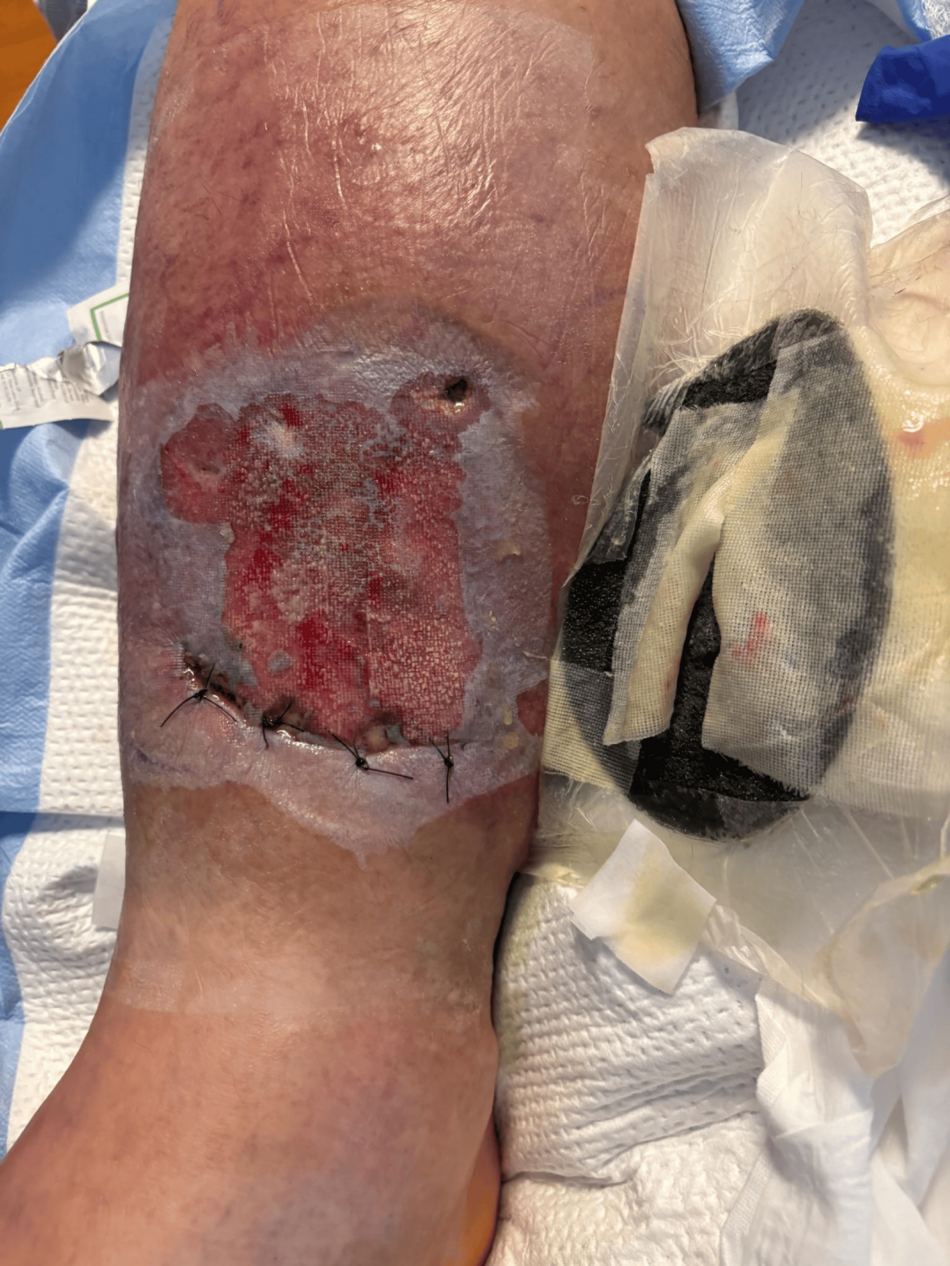
Patient 2: day eight of hospitalization. Postoperative day one after surgical debridement.

Initially, the clinical presentation was thought to be consistent with cellulitis. He was empirically started on intravenous cefepime before being admitted for inpatient care. Preliminary blood culture reports indicated the presence of Gram-negative rods; subsequently, the patient was started on doxycycline, ceftazidime, and metronidazole. Final blood cultures tested via MALD/TOF resulted on hospital day three, confirming the presence of *V. vulnificus* bacteremia. At that time, surgery was consulted for debridement, which was performed the following day. Figure 8 and Figure 9 show the wound before debridement, and Figure 10 shows the wound immediately after. Wound cultures of the left lower extremity were also obtained, which grew *Staphylococcus aureus*. Intravenous vancomycin was started on hospital day two.

Intravenous antibiotic therapy was initiated during the inpatient hospitalization and continued at the rehabilitation facility, totaling a four-week course of intravenous treatment. At the time of discharge, the patient was afebrile, hemodynamically stable, and demonstrating improvement of the affected extremity. He was discharged to a rehabilitation center for ongoing wound care and completion of antibiotic therapy, with outpatient infectious disease follow-up.

## Discussion

*V. vulnificus* is a Gram-negative, motile, curved rod-shaped bacterium that belongs to the Vibrionaceae family. It is a facultative anaerobe and halophilic, requiring salt for optimal growth [5]. The surface of CI is enveloped by a capsular polysaccharide (CPS); structural variation in the CPS shields the bacterium from complement-mediated lysis and other innate immune defenses, limiting membrane-attack-complex deposition and thereby promoting immune evasion and enhancing virulence [6].

*V. vulnificus* thrives in warm marine environments, particularly at temperatures above 20°C, with increased prevalence in warmer waters [7]. Incidence peaks between July and October when sea surface temperatures exceed 30°C [8]. Post‑hurricane surges are well recognized; following Hurricane Ian in 2022, the incidence of *V. vulnificus* increased by approximately 1,100% based on historical surveillance, most of which were caused by *V. vulnificus* [9].

Symptoms of *V. vulnificus* infections depend on exposure. Gastrointestinal infections present with diarrhea, vomiting, and abdominal pain. Wound infections can progress rapidly to necrotizing fasciitis, hemorrhagic bullae, and tissue necrosis. In immunocompromised individuals, the bacterium can enter the bloodstream, leading to sepsis and multi-organ failure [10].

*V. vulnificus* can enter the body through ingestion of raw or undercooked seafood or by wound exposure to contaminated water. Gastrointestinal infections can progress to primary septicemia, particularly in immunocompromised individuals. Bloodstream infections can trigger disseminated intravascular coagulation, a widespread microvascular thrombosis, which leads to characteristic hemorrhagic skin lesions known as purpura fulminans. Purpura fulminans is a rapidly progressive, hemorrhagic dermatosis with a high mortality rate, in some cases nearing 100% [11].

Once inside the body, *V. vulnificus* adheres to host cells using pili and outer membrane proteins. It evades immune responses through capsule formation; encapsulation enhances resistance to phagocytosis, making systemic infection more likely [12]. *V. vulnificus* produces a variety of toxins; hemolysins and cytotoxins facilitate cell lysis, increasing vascular permeability, which contributes to the formation of hemorrhagic bullae [6]. The metalloprotease VvpE degrades host proteins, aiding in tissue invasion and necrosis [13]. Additionally, the bacterium produces siderophores, which scavenge iron, enhancing its survival and increasing the risk of sepsis [14].

Early antimicrobial therapy is essential because fatality rises if antibiotics are delayed beyond 24 hours [15]. Current Centers for Disease Control and Prevention guidance recommends combination therapy with a tetracycline such as doxycycline or minocycline in combination with a third‑generation cephalosporin such as ceftazidime or ceftriaxone [3]. A treatment duration of 10-14 days suffices for uncomplicated bacteremia, whereas necrotizing soft tissue infection warrants at least 21 days, tailored to clinical response. Early surgical consultation is imperative; debridement or amputation undertaken within 12 hours of diagnosis significantly reduces mortality [16].

## Conclusions

These cases emphasize the increasing prevalence of *V. vulnificus*. With future climate predictions stating further increases in global temperatures, cases can only be expected to rise. Given the increasing incidence of *V. vulnificus* infections due to more frequent and adverse hurricanes, physicians and healthcare providers must maintain a high index of suspicion for *Vibrio* bacteremia in patients presenting with recent exposure to floodwaters. Early recognition is essential, as *Vibrio* bacteremia can progress rapidly, leading to significant morbidity, loss of limb, and even death. As demonstrated in our cases, prompt identification and immediate intervention are crucial in managing the infection and preventing severe outcomes.
